# The entropy solution of a hyperbolic-parabolic mixed type equation

**DOI:** 10.1186/s40064-016-3374-z

**Published:** 2016-10-18

**Authors:** Huashui Zhan

**Affiliations:** School of Applied Mathematics, Xiamen University of Technology, Xiamen, 361024 China

**Keywords:** Hyperbolic-parabolic mixed type equation, $n-$dimensional cube, Entropy solution, Kruzkov’s bi-variables method, 35L65, 35K85, 35R35

## Abstract

The entropy solution of the equation

$$\frac{\partial u}{\partial t} = \Delta A(u) +\text {div}(b(u)),\ \ (x,t)\in \Omega \times (0,T),$$

is considered. Besides the usual initial value, only a partial boundary value is imposed. By choosing some special test functions, the stability of the solutions is obtained by Kruzkov’s bi-variables method, provided that $\Omega \subset \mathbb {R}^{N}$ is an unit *n*-dimensional cube or the half space.

## The boundary condition

We consider the equation

1 $$\frac{\partial u}{\partial t} =\Delta A(u) +\text {div}(b(u)),\ \ (x,t)\in \Omega \times (0,T),$$

and assume that

2 $$A(u)=\int _{0}^{u}a(s)ds,\;\;a(s)\ge 0, a(0)=0,$$

where $\Omega \subset \mathbb {R}^{N}$ is a appropriately smooth open domain. Equation (1) is with hyperbolic-parabolic mixed type, arises from the reaction diffusion problem (Wu et al. 2001), the stationary boundary layer theory (Oleinik and Samokhin 1999).

For Cauchy problem of Eq. (1), the paper (Vol’pert and Hudjaev 1967) was the first one to study its solvability, since then, many papers continued to dedicate to the problem, many excellent results were obtained, one can refer to Wu et al. (2001) and Refs. Bendahamane and Karlsen (2004), Brezis and Crandall (1979), Carrillo (1999), Chen and Dibenedetto (2001), Chen and Perthame (2003), Cockburn and Gripenberg (1999), Evans (1998), Karlsen and Risebro (2003), Kružkov (1970), Oleinik and Samokhin (1999), Vol’pert (1967), Vol’pert and Hudjaev (1967); Vol’pert and Hudjave (1975), Wu and Yin (1989), Wu et al. (2001), Zhan (2004), Zhao (1985), Zhao and Zhan (2005).

If we want to consider the initial boundary value problem of Eq. (1), the initial value is always imposed

3 $$u(x,0)=u_{0}(x), \quad x\in \Omega .$$

But can we impose Dirichlet homogeneous boundary value

4 $$u(x,t)=0,\ (x,t)\in \partial \Omega \times (0,T),$$

as usual? When the equation is of weakly degenerate, i.e. there is not interior point in the set $\{s: a(s)=0\}$, we can impose Dirichlet homogeneous boundary condition (4). One can refer to Wu et al. (2001) and the references therein. When the equation is of strongly degenerate, i.e. there is an interior point in the set $\{s: a(s)=0\}$, there are two ways to deal with the corresponding problem. In one way, the entropy solution *u* is a *BV* function, which means that

$$\iint_{Q_T}\left|\frac{\partial u}{\partial t}\right|dxdt\le c,\quad \iint_{Q_T}\left|\frac{\partial u}{\partial x_{i}}\right|dxdt\le c,\quad i=1,2, \ldots , N.$$

It is well-known that the *BV* function is the weakest function that one can define the trace on the boundary. In this way, we can directly answer whether (4) is true or not in the sense of the trace, and the general result is that, instead of (4), only a partial boundary value such as

5 $$u(x,t)=0,\ (x,t)\in \Sigma _{1} \times (0,T),$$

is imposed, where $\Sigma _{1}\subseteq \partial \Omega$ is a relative open subset of $\partial \Omega$. The representative works by Wu and Zhao (1983a, b) had been accomplished in early 1980s, later, one can refer to Yin and Wang (2007). In the other way, the boundary value condition is not directly shown in the sense of the trace as (4), but is elegantly implicitly contained in a family entropy inequalities. Moreover, the entropy solutions defined in this way are only in $L^{\infty }$ space, the existence of the traditional trace [which was called the strong trace in Kobayasi and Ohwa (2012)] on the boundary is not guaranteed, so the boundary value condition is satisfied in a weaker sense than the sense of the trace, one can refer to Carrillo (1999), Li and Qin (2012), Lions et al. (1994), Kobayasi and Ohwa (2012) for more details.

Recently, by the parabolic regularization method, the author Zhan (2015a) had shown the explicit formula of $\Sigma _1$ in (5). Let us give some details.

For small $\eta >0$, let

$$S_{\eta }(s)=\int _{0}^{s}h_{\eta }(\tau )d\tau , \ h_{\eta }(s)=\frac{2}{\eta }\left( 1-\frac{|s|}{\eta }\right)_{+}.$$

Obviously $$h_{\eta }(s)\in C(\mathbb {R})$$, and

6 $$h_{\eta }(s)\ge 0,\;\ |{sh}_{\eta}(s)| \le 1,\;|S_{\eta }(s)| \le 1;\underset{\eta \rightarrow 0}{\lim }S_{\eta }(s)=\text {sgn}s,\;\underset{\eta \rightarrow 0}{\lim }sS_{\eta }^{\prime }(s)=0.$$

Let

7 $$\Sigma _{1\eta k}= \left\{ x\in \Sigma ,\ S_{\eta }(k)[b_{i}(0)-b_{i}(k)]n_{i}(x)>0\right\} ,$$

8 $$\Sigma _{2\eta k}= \left\{ x\in \Sigma ,\ S_{\eta }(k)[b_{i}(0)-b_{i}(k)]n_{i}(x)\le 0\right\} .$$

Here and in what follows, $\{n_{i}\}_{i=1}^{N}$ is the inner normal vector of $\Omega$. Clearly, $\partial \Omega =\Sigma =\Sigma _{1\eta k}\bigcup \Sigma _{2\eta k}$. Then

9 $$\Sigma _{1}=\bigcup _{\forall \eta \ge 0,\forall k\in \mathbb {R}}\Sigma _{1\eta k},\ \ \Sigma _{2}=\Sigma \backslash \Sigma _{1}.$$

Basing on (9), if the domain $\Omega$ is bounded, the existence of the entropy solution had been proved in Zhan (2015a). Assuming that

10 $$|\triangle d|\le c,\quad \ \frac{1}{\lambda }\int _{\Omega _{\lambda }}dxdt\le c,$$

the stability of the solutions also had been proved in Zhan (2015a). Here $d(x)=dist(x,\partial \Omega )$, and $\Omega _{\lambda }=\{x\in \Omega , d(x)<\lambda \}$, when $\lambda$ is small enough. If the domain $\Omega =\mathbb {R}^{N}_{+}$ is the half space of $\mathbb {R}^{N}$, recently in Zhan (2015b), the author had shown that if $b'_{N}(0)<0$, then, $\Sigma _{1}=\partial \mathbb {R}^{N}_{+}$, we can impose Dirichlet boundary value

$$u(x,t)=0,\ (x,t)\in \partial \mathbb {R}^{N}_{+} \times (0,T).$$

But if $b'_{N}(0)\ge 0$, then, $\Sigma _{1}=\emptyset$, no any boundary value condition is necessary, the solution of the equation is free from any limitation of the boundary value condition.

Now, inspired by Zhan (2004, 2015a, b) and Zhao and Zhan (2005), we give a new definition of the entropy solution.

### **Definition 1**

 A function *u* is said to be the entropy solution of Eq. (1) with the initial value (3) and with the partial boundary value (5), if

1. *u* satisfies
  $$u\in BV(Q_{T})\cap L^{\infty }(Q_{T}),\quad \frac{\partial }{\partial x_{i}}\int _{0}^{u}\sqrt{a(s)}ds\in L^{2}(Q_{T}).$$
2. For any $\varphi \in C_{0}^{2}(Q_{T})$, $\varphi \ge 0$, for any $k\in \mathbb {R}$, for any small $\eta >0$, *u* satisfies
  11 $$\iint\nolimits _{Q_{T}}\left[ I_{\eta }(u-k)\varphi_{t}-B_{\eta }^{i}(u,k)\varphi _{x_{i}}+A_{\eta }(u,k)\Delta \varphi-S_{\eta }^{\prime }(u-k)\left| \nabla \int _{0}^{u}\sqrt{a(s)}ds\right|^{2}\varphi \right] dxdt\ge 0,$$
  where
  $$B_{\eta }^{i}(u,k)=\int _{k}^{u}b_{i}^{\prime }(s)S_{\eta }(s-k)ds,\;A_{\eta }(u, k)=\int _{k}^{u}a(s)S_{\eta }(s-k)ds,\ I_{\eta }(u-k)=\int _{0}^{u-k}S_{\eta }(s)ds.$$
3. The homogeneous boundary value (5) is satisfied in the sense of that
  12 $$\gamma u\mid _{\Sigma _{1\eta k}}=0,$$
  for any $k, \eta$. Here $\gamma u$ means that the equality is true in the sense of the trace.
4. If the domain $\Omega$ is bounded, the initial value is true in the sense that
  13 $$\ \ \ \underset{t\rightarrow 0}{\lim }\int _{\Omega }\left| u(x,t)-u_{0}(x)\right| dx=0,\;\;\text {a.e.}\quad x\in \Omega . \;$$
  If the domain is unbounded, the initial value is true in the sense that
  $$\ \ \ \underset{t\rightarrow 0}{\lim }\int _{\Omega _{R} }\left|u(x,t)-u_{0}(x)\right| dx=0,\;\;\text {a.e.}\quad x\in \Omega , \qquad \qquad \qquad (13)'$$
  where $$\Omega _{R}=B(0,R)\bigcap \Omega.$$
  The existence of the entropy solution in the sense of Definition  1 can be proved similar as that in Zhan (2015a), we omit the details here. In our paper, we are mainly concern with the stability of the entropy solutions of Eq. (1) without the condition (10). For simplicity, only some special domains, for examples, the unite $n-$dimensilnal cube
  $$D_{1}=\{(x_1, x_2, \ldots , x_N): 0<x_i<1, i=1, 2, \ldots , N \}.$$
  and the half space $\mathbb {R}^{N}_{+}$, are considered. By choosing special test functions, we will prove the following theorem.

### **Theorem 1**

*Suppose that* *A*(*s*) *and* $b_{i}(s)$ *are smooth enough,* $\Omega =D_{1}$ *is the unite* $n-$ *dimensilnal cube. If* $\Sigma _{1}$ *is a subset of* $\Sigma$, *let* *u*, *v* *be solutions of Eq.* (1) *with the different initial values* $u_{0}(x)$, $v_{0}(x)\in L^{\infty }(\Omega )$ *respectively. Suppose that*

14 $$\gamma u(x,t)=f(x,t),\ \gamma v=g(x,t),\ (x,t)\in \Sigma \times (0,T),$$

*and in particular,*

15 $$\gamma u=\gamma v=0,\ x\in \Sigma _{1}.$$

*Then*

16 $$\int _{\Omega }\left| u(x,t)-v(x,t)\right| dx\le \int _{\Omega }\left| u_{0}-v_{0}\right| dx+{\mathrm{ess}}\sup _{(x,t)\in \Sigma _{2}\times (0,T)}|f\mathrm (x,t\mathrm )-g\mathrm (x,t\mathrm )|,$$

*where* $(x,t)\in \mathbb {R}^{N+1}$, $\mathrm {ess}\sup _{(x\mathrm ,t\mathrm )\in \Sigma _{2}\times (0,T)}|f\mathrm (x,t\mathrm )-g\mathrm (x,t\mathrm )|$ *is in the sense of* $N-$ *dimensonal Hausdorff measure.*

Compared Theorem  1 to the results obtained in Zhan (2015a), the essential innovation lies in that, without the condition (10), by skillfully constructing the testing function, we still can obtain the stability. At the last section, we also study the similar problem on half space $\mathbb {R}^{N}_{+}$ and get the similar result, this result is just the same as that in Zhan (2015b), but we supply a simpler proof.

Now, let us give some analysis in the boundary value condition (5) or (12) to see the rationality. By the definition of $\Sigma _{1\eta k}$, we know that

$$0<S_{\eta }(k)[b_{i}(0)-b_{i}(k)]n_{i}=-kS_{\eta }(k)b_{i}'(\zeta )n_{i},\quad x\in \Sigma$$

where $\zeta \in (k,0)$. If we let $\eta \rightarrow 0$. Then

$$b_{i}'(\zeta )n_{i}(x)<0,\quad x\in \Sigma.$$

Let $k\rightarrow 0$. We have

17 $$b_{i}'(0)n_{i}(x)<0,\quad x\in \Sigma .$$

The last inequality (17) is in according with the classical Fichrea–Oleinik theory, one can refer to the explanation in previous works (Zhan 2015a, b)

Let us come back our definition. On the unite $n-$dimensilnal cube $D_1$, according to the homogeneous boundary value condition (5), and by (17), we have

18 $$\gamma u=\gamma v=0, x\in \bigcup _{i=1}^{N}\left\{ x\in \partial D_{1}: \left( x_{i}-\frac{1}{2}\right) b_{i}'(0)>0\right\} .$$

For example, when we consider the boundary plane $\{x_N=0\}$, $\mathbf {n}=\{0,0,\ldots , 0,1\}$, (17) implies $b_{i}'(0)n_{i}=b_{N}'(0)<0$, (18) is true on $\{x_N=0\}$, we should give the boundary value on $\{x_N=0\}$, while, on $\{x_N=1\}$ we can not give the boundary value. If $b_{N}'(0)>0$, then (18) is true on $\{x_N=1\}$, which implies that we should give the boundary value on $\{x_N=1\}$, while, on $\{x_N=0\}$ we can not give the boundary value. Certainly, if $b_{N}'(0)=0$, both on $\{x_N=0\}$ and on $\{x_N=1\}$, no boundary value condition is imposed.

## Kruzkov’s bi-variables method

Let $\Gamma _{u}$ be the set of all jump points of $u\in BV(Q_{T}),v$ the normal of $\Gamma _{u}$ at $X=(x,t)$, $u^{+}(X)$ and $u^{-}(X)$ the approximate limits of *u* at $X\in \Gamma _{u}$ with respect to $(v,Y-X)>0$ and $(v,Y-X)<0$ respectively. For continuous function *p*(*u*, *x*, *t*) and $u\in BV(Q_{T})$, define

19 $$\widehat{p}(u,x,t)=\int _{0}^{1}p(\tau u^{+}+(1-\tau )u^{-},x,t)d\tau,$$

which is called the composite mean value of *p*. For a given *t*, we denote $\Gamma _{u}^{t},\;H^{t},(v_{1}^{t},\;\ldots ,v_{N}^{t})$ and $u_{\pm }^{t}$ as all jump points of $u(\cdot ,t)$, Housdorff measure of $\Gamma _{u}^{t}$, the unit normal vector of $\Gamma _{u}^{t}$, and the asymptotic limit of $u(\cdot ,t)$ respectively. Moreover, if $f(s)\in C^{1}( \mathbb {R}),\;u\in BV(Q_{T})$, then $f(u)\in BV(Q_{T})$ and

20 $$\frac{\partial f(u)}{\partial x_{i}}=\widehat{f^{\prime }}(u)\frac{\partial u}{\partial x_{i}},\quad i=1, 2, \ldots , N, N+1,$$

where $x_{N+1}=t$ as usual.

### **Lemma 1**

*Let* *u* *be the solution of Eq.* (1) *in the sense of Definition*  1. *Then*

21 $$a(s)=0,\;s\in I(u^{+}(x,t),u^{-}(x,t))\;\;a.e.\;on\;\Gamma _{u},$$

*which* $I(\alpha ,\beta )$ *denote the closed interval with endpoints* $\alpha$ *and* $\beta$, *and *(21) *is in the sense of Hausdorff measure* $H_{N}(\Gamma _{u})$.

This lemma can be proved in a similar way as Zhan (2004); Zhao and Zhan (2005), we omit the details here.

Now, we will show that how Kruzkov’s bi-variables method, which was used to deal with the conservation law equation (Kružkov 1970) originally, can be used to prove the stability of the solutions to Eq. (1). Let *u*, *v* be two entropy solutions of Eq. (1) with initial values

$$u(x,0)=u_{0}(x),\;v(x,0)=v_{0}(x),$$

and with the boundary values (14)–(15), in particular, $u(x,t)=v(x,t)=0, (x,t)\in \Sigma _{1}\times (0,T)$.

By Definition  1, for $\varphi \in C_{0}^{2}(Q_{T})$, we have

22 $$\iint\nolimits _{Q_{T}}\left[ I_{\eta }(u-k)\varphi _{t}-B_{\eta }^{i}(u,k)\varphi _{x_{i}}+A_{\eta }(u,k)\Delta \varphi -S_{\eta }^{\prime }(u-k)\left|\nabla \int _{0}^{u}\sqrt{a(s)}ds\right|^{2}\varphi \right] dxdt\ge 0,$$

23 $$\iint\nolimits _{Q_{T}}\left[ I_{\eta }(v-l)\varphi _{\tau }-B_{\eta }^{i}(v,l)\varphi _{y_{i}}+A_{\eta }(v,l)\Delta \varphi -S_{\eta }^{\prime }(v-l)\left| \nabla \int _{0}^{v}\sqrt{a(s)}ds\right|^{2}\varphi \right] dyd\tau \ge 0.$$

Let $\psi (x,t,y,\tau )=\phi (x,t)j_{h}(x-y,t-\tau )$, where $\phi (x,t)\ge 0,\;\phi (x,t)\in C_{0}^{\infty }(Q_{T})$, and

24 $$j_{h}(x-y,t-\tau )=\omega _{h}(t-\tau )\Pi _{i=1}^{N}\omega _{h}(x_{i}-y_{i}).$$

Here

25 $$\omega _{h}(s)=\frac{1}{h}\omega \left( \frac{s}{h}\right) ,\omega (s)\in C_{0}^{\infty }(R),\;\omega (s)\ge 0,\;\omega (s)=0\;if\;\left|s\right| >1,\;\int _{-\infty }^{\infty }\omega (s)ds=1.$$

We choose $k=v(y,\tau ),\;l=u(x,t),\;\varphi =\psi (x,t,y,\tau )$ in (22) (23), integrate over $Q_{T}$, then

26 $$\iint \nolimits _{Q_{T}}\iint \nolimits _{Q_{T}}\left[ I_{\eta }(u-v)(\psi _{t}+\psi _{\tau })-(B_{\eta }^{i}(u,v)\psi _{x_{i}}+B_{\eta }^{i}(v,u)\psi _{y_{i}})+A_{\eta }(u,v)\Delta _{x}\psi +A_{\eta }(v,u)\Delta _{y}\psi \right] -S_{\eta }^{\prime }(u-v)\left( \left|\nabla \int _{0}^{u}\sqrt{a(s)}ds\right|^{2}+\left|\nabla \int _{0}^{v}\sqrt{a(s)}ds\right|^{2}\right) \psi dxdtdyd\tau =0.$$

Clearly,

$$\frac{\partial j_{h}}{\partial t}+\frac{\partial j_{h}}{\partial \tau }=0,\quad \frac{\partial j_{h}}{\partial x_{i}}+\frac{\partial j_{h}}{\partial y_{i}}=0,\quad i=1,\ldots ,N;\quad \frac{\partial \psi }{\partial t}+\frac{\partial \psi }{\partial \tau }=\frac{\partial \phi }{\partial t}j_{h},\quad \frac{\partial \psi }{\partial x_{i}}+\frac{\partial \psi }{\partial y_{i}}=\frac{\partial \phi }{\partial x_{i}}j_{h}.$$

Noticing that

$$\underset{\eta \rightarrow 0}{\lim }B_{\eta }^{i}(u,v)=\underset{\eta \rightarrow 0}{\lim }B_{\eta }^{i}(v,u)=\text {sgn}(u-v)(b_{i}(u)-b_{i}(v)),$$

as $\eta \rightarrow 0$, we have,

$$\iint_{Q_{T}}\iint_{Q_{T}}[B_{\eta }^{i}(u,v)\psi _{x_{i}}+ B_{\eta }^{i}(v,u)\psi _{y_{i}}]dxdtdyd\tau \rightarrow \iint_{Q_{T}}\iint_{Q_{T}}\text {sgn}(u-v)[b_{i}(u)-b_{i}(v)]\phi _{x_{i}}j_{h}dxdtdyd\tau ,$$

as $h\rightarrow 0$, we have

27 $$\begin{gathered} \iint_{{Q_{T} }} {\iint_{{Q_{T} }} {{\text{sgn}}}}(u - v)[b_{i} (u) - b_{i} (v)]\phi _{{x_{i} }} j_{h} dxdtdyd\tau \hfill \\ \quad \to \iint_{{Q_{T} }} {{\text{sgn}}}(u - v)[b_{i} (u) - b_{i} (v)]\phi _{{x_{i} }} dxdt. \hfill \\ \end{gathered}$$

At the same time, we have

28 $$\iint_{Q_{T}}\iint _{Q_{T}}S_{\eta }^{\prime }(u-v)\left(\left|\nabla_{x}\int_{0}^{u}\sqrt{a(s)}ds\right|^{2}+\left|\nabla_{y}\int_{0}^{v}\sqrt{a(s)}ds\right|^{2}\right) \psi dxdtdyd\tau =\iint_{Q_{T}}\iint_{Q_{T}}S_{\eta}^{\prime }(u-v)\left( \left|\nabla_{x}\int_{0}^{u}\sqrt{a(s)}ds\right|-\left|\nabla_{y}\int_{0}^{v}\sqrt{a(s)}ds\right|\right) ^{2}\psi dxdtdyd\tau +2\iint_{Q_{T}}\iint_{Q_{T}}S_{\eta}^{\prime}(u-v)\nabla_{x}\int_{0}^{u} \sqrt{a(s)}ds\cdot\nabla_{y}\int_{0}^{v}\sqrt{a(s)}ds\psi dxdtdyd\tau,$$

and

29 $$\iint_{Q_{T}}[A_{\eta }(u,v)\Delta _{x}\psi +A_{\eta }(v,u)\Delta _{y}\psi ]dxdtdyd\tau =\iint_{Q_{T}}\iint_{Q_{T}}\{A_{\eta }(u,v)(\Delta _{x}\phi j_{h}+2\phi _{x_{i}}j_{hx_{i}}+\phi \Delta j_{h})+A_{\eta }(v,u)\phi \Delta _{y}j_{h}\}dxdtdyd\tau =\iint_{Q_{T}}\iint_{Q_{T}}\{A_{\eta }(u,v)\Delta _{x}\phi j_{h}+A_{\eta }(u,v)\phi _{x_{i}}j_{hx_{i}}+A_{\eta }(v,u)\phi _{x_{i}}j_{hy_{i}}\}dxdtdyd\tau -\iint_{Q_{T}}\iint_{Q_{T}}\{\widehat{a(u)S_{\eta }(u-v)}\frac{\partial u}{ \partial x_{i}}-\widehat{\int _{u}^{v}a(s)S_{\eta }^{\prime }(s-v)ds}\frac{ \partial u}{\partial x_{i}})\phi j_{hx_{i}}\}dxdtdyd\tau,$$

where

$$\widehat{a(u)S_{\eta }(u-v)}=\int _{0}^{1}a(su^{+}+(1-s)u^{-})S_{\eta }(su^{+}+(1-s)u^{-}-v)ds, \int _{u}^{v}\widehat{a(s)S_{\eta }^{\prime }(s-v)}ds=\int _{0}^{1} \int _{su^{+}+(1-s)u^{-}}^{v}a(\sigma )S_{\eta }(\sigma -su^{+}-(1-s)u^{-})d\sigma ds.$$

Now, we will combine the last term on the right hand of (28) with the last term on the right hand side of (29). In details, by Lemma  1, at one hand, we have

30 $$\iint_{Q_{T}} \iint_{Q_{T}}\nabla_{x}\nabla_{y}\int_{v}^{u} \sqrt{a(\delta)}\int_{\delta}^{v}\sqrt{a(\sigma)}S_{\eta }^{\prime}(\sigma-\delta)d\sigma d\delta \psi dxdtdyd \tau =\iint_{Q_{T}}\iint_{Q_{T}}\int _{0}^{1}\int _{0}^{1}\sqrt{ a(su^{+}+(1-s)u^{-})}\sqrt{a(\sigma v^{+}+(1-\sigma )v^{-})} \times S_{\eta }^{\prime }[\sigma v^{+}+(1-\sigma )v^{-}-su^{+}-(1-s)u^{-}]dsd\sigma \nabla _{x}u\nabla _{y}vdxdtdyd\tau =\iint_{Q_{T}}\iint_{Q_{T}}\int _{0}^{1}\int _{0}^{1}S_{\eta }^{\prime }[\sigma v^{+}+(1-\sigma )v^{-}-su^{+}-(1-s)u^{-}]dsd\sigma \widehat{\sqrt{a(u)}}\nabla _{x}u\widehat{\sqrt{a(v)}}\nabla _{y}vdxdtdyd\tau =\iint_{Q_{T}}\iint_{Q_{T}}\int _{0}^{1}\int _{0}^{1}S_{\eta }^{\prime }(v-u)\nabla _{x}\int _{0}^{u}\sqrt{a(s)}ds\nabla _{y}\int _{0}^{v}\sqrt{a(s)}dsdxdtdyd\tau .$$

At the other hand, we have

31 $$\iint_{Q_{T}}\iint_{Q_{T}}\nabla _{x}\nabla _{y}\int _{v}^{u}\sqrt{a(\delta )}\int _{\delta }^{v}\sqrt{a(\sigma )}S_{\eta }^{\prime }(\sigma -\delta )d\sigma d\delta \psi dxdtdyd\tau =\iint_{Q_{T}}\iint_{Q_{T}}\int _{0}^{1}\sqrt{a(su^{+}+(1-s)u^{-})} \times \int _{su^{+}+(1-s)u^{-}}^{v}\sqrt{a(\sigma )}S_{\eta }^{\prime }(\sigma -su^{+}-(1-s)u^{-})d\sigma ds\frac{\partial u}{\partial x_{i}}j_{hx_{i}}\phi dxdtdyd\tau .$$

By (30) (31), we have

32 $$\iint_{Q_{T}}\iint_{Q_{T}}\left(\widehat{a(u)S_{\eta}(u-v)}\frac{\partial u}{\partial x_{i}}-\widehat{\int_{u}^{v}a(s)S_{\eta }^{\prime }(s-u)ds}\frac{\partial u}{\partial x_{i}}\right)j_{hx_{i}}\phi dxdtdyd\tau +2\iint_{Q_{T}}\iint_{Q_{T}}S_{\eta }^{\prime }(u-v)\nabla _{x}\int _{0}^{u} \sqrt{a(s)}ds\cdot \nabla _{y}\int _{0}^{v}\sqrt{a(s)}ds\psi dxdtdyd\tau =\iint_{Q_{T}}\iint_{Q_{T}}\left[\int _{0}^{1}a(su^{+}+(1-s)u^{-})S_{\eta }(su^{+}+(1-s)u^{-}-v)ds\nonumber -\int _{0}^{1}\int _{su^{+}+(1-s)u^{-}}^{v}a(\sigma )S_{\eta }^{\prime }(\sigma -su^{+}-(1-s)u^{-})d\sigma ds\nonumber +2\int _{0}^{1}\sqrt{a(su^{+}+(1-s)u^{-})}\int _{su^{+}+(1-s)u^{-}}^{v} \sqrt{a(\sigma )}S_{\eta }^{\prime }(\sigma -su^{+}-(1-s)u^{-})d\sigma ds\right]\frac{\partial u}{\partial x_{i}}j_{hx_{i}}\phi dxdtdyd\tau \nonumber =-\iint_{Q_{T}}\iint_{Q_{T}}\int _{0}^{1}\int _{su^{+}+(1-s)u^{-}}^{v}\left[ \sqrt{a(\sigma )}-\sqrt{a(su^{+}+(1-s)u^{-})}\right]\nonumber \times S^{\prime }_{\eta } (\sigma -su^{+}-(1-s)u^{-})d\sigma ds\frac{\partial u}{ \partial x_{i}}j_{hx_{i}}\phi dxdtdyd\tau \rightarrow 0,$$

as $$\eta \rightarrow 0$$.

Let us come back (29). Since

$$\lim _{\eta \rightarrow 0}A_{\eta }(u,v)=\underset{\eta \rightarrow 0}{\lim }A_{\eta }(v,u)=\text {sgn}(u-v)[A(u)-A(v)],$$

we have

33 $$\lim _{\eta \rightarrow 0}[A_{\eta }(u,v)\phi _{x_{i}}j_{hx_{i}}+A_{\eta }(u,v)\phi _{y_{i}}j_{hy_{i}}]=0.$$

Combing (26)–(28) with (32)–(33), and letting $\eta \rightarrow 0,h\rightarrow 0$ in (26). We obtain

34 $$\iint_{Q_{T}}\left[\left|u(x,t)-v(x,t)\right|\phi _{t}-\text {sgn} (u-v)(b_{i}(u)-b_{i}(v))\phi _{x_{i}}+ |A(u)-A(v)|\Delta \phi \right] dxdt = \iint_{Q_{T}}\iint_{Q_{T}}S_{\eta }^{\prime }(u-v)\left(\left|\nabla _{x}\int _{0}^{u}\sqrt{a(s)}ds\right|-\left|\nabla _{y}\int _{0}^{v}\sqrt{a(s)}ds\right|\right) ^{2}\psi dxdtdyd\tau \ge 0.$$

By Kruzkov’s bi-variables method it means that, by a process of limit, we can choose a suitable test function $\phi \in C^1_0(Q_T)$ in (34), to obtain the stability of the solutions.

## Proof of Theorem  1

*The proof of Theorem  1* Let $x=\{x_1, x_2, \ldots , x_i, \ldots , N\}$ and define

35 $$d_i(x_{i})=\left\{ \begin{array}{ll} x_i,\ \ &{}\ \ \mathrm{if}\ \ 0\le x_i\le \frac{1}{2},\\ 1-x_i,\ \ &{}\ \ \mathrm{if}\ \frac{1}{2}<x_i\le 1. \end{array}\right.$$

For small enough $\lambda$, we set

36 $$\varphi _{i\lambda }(x_{i})=\left\{ \begin{array}{cc} \sin \frac{1}{\lambda }(d_i(x_i)),\ \ & \mathrm{if}\ \ 0\le d_i(x_i)\le \frac{\pi \lambda }{2},\\ 1,\ \ & \mathrm{if}\ d_i(x)\ge \frac{\pi \lambda }{2}. \end{array} \right.$$

Let $0\le \eta (t)\in C_0^{1}(t)$ and choose the test function in (34) as

37 $$\phi (x,t)=\eta (t)\prod _{j=1}^{N}\varphi _{j\lambda }(x_{j}).$$

Then

38 $$\partial _{x_{i}}\phi (x,t)=\eta (t)\partial _{x_{i}}\varphi _{i\lambda }(x_{i})\prod _{j=1,j\ne i}^{N}\varphi _{j\lambda }(x_{j}) =\eta (t)\frac{1}{\lambda }\cos \frac{1}{\lambda }(d_i(x_i))d_{ix_i}(x_{i})\prod _{j=1,j\ne i}^{N}\varphi _{j\lambda }(x_{j}), \ \ \ 0\le d_i(x_i)\le \frac{\pi \lambda }{2}.$$

39 $$\triangle \phi (x,t)=\frac{1}{\lambda }\eta (t)\prod _{j=1,j\ne i}^{N}\varphi _{j\lambda }(x_{j}) \left[ -\frac{1}{\lambda }\sin \frac{1}{\lambda }(d_i(x_i))d_{ix_{i}}^{2}+\frac{1}{\lambda }\cos \frac{1}{\lambda }(d_i(x_i))\triangle d_{i}(x_i)\right] =-\frac{1}{\lambda ^{2}}\eta (t)\prod _{j=1,j\ne i}^{N}\varphi _{j\lambda }(x_{j}) \sin \frac{1}{\lambda }(d_i(x_i))d_{ix_{i}}^{2}\le 0,\ \ 0\le d_i(x_i)\le \frac{\pi \lambda }{2}.$$

For (39) in (34),

40 $$\iint_{Q_{T}}\left[ \left|u(x,t)-v(x,t)\right|\phi _{t}-\text {sgn} (u-v)(b_{i}(u)-b_{i}(v))\partial _{x_{i}}\phi (x,t)\right] dxdt\ge 0.$$

By that $|\partial _{x_{i}}\phi (x,t)|\le \frac{1}{\lambda }$, $|b_{i}(u)-b_{i}(v)|\le c|u-v|$, we have

41 $$\iint_{Q_{T}}|u(x,t)-v(x,t)|\phi _{t}dxdt +c\int _{0}^{T}\int _{\Omega _{\lambda }}\eta (t)\frac{1}{\lambda }|\left| u-v\right|dxdt\ge 0,$$

where $\Omega _{\lambda }=\{x\in \Omega : d_i(x_i)<\frac{\lambda \pi }{2}\}$. According to the definition of the trace of BV functions (Enrico 1984), when $x\in \Sigma _{1}$, $\gamma u=\gamma v=0$, let $\lambda \rightarrow 0$ in (41). We have

$$\lim_{\lambda \rightarrow 0}\int_{0}^{T}\eta(t)\frac{1}{\lambda }\int _{\Omega _{\lambda }}|\left|u-v\right| dxdt=c\int _{0}^{T}\eta (t)|u-v|_{\partial \Omega }dt =c\int _{0}^{T}\eta (t)|u-v|_{\partial \Omega }dt\le c\cdot \text {essup}_{\Sigma _{2}\times (0,T)}|f(x,t)-g(x,t)|.$$

Let $\lambda \rightarrow 0$ in (41). Then

42 $$c\cdot \text {essup}_{\Sigma _{2}\times (0,T)}|f(x,t)-g(x,t)|+\iint _{Q_{T}}\mid u(x,t)-v(x,t)\mid \eta '_{t}dxdt\ge 0.$$

Let $$0<s<\tau <T$$, and

$$\eta (t)=\int _{\tau -t}^{s-t}\alpha _{\varepsilon }(\sigma )d\sigma ,\;\ \varepsilon <\min \{\tau , T-s\}.$$

Here $\alpha _{\varepsilon }(t)$ is the kernel of mollifier with $\alpha _{\varepsilon }(t)=0$ for $t\notin (-\varepsilon ,\varepsilon )$. Then

$$c\cdot \text {essup}_{\Sigma _{2}\times (0,T)}|f(x,t)-g(x,t)| +\int _{0}^{T}\left[ \alpha _{\varepsilon }(t-s)-\alpha _{\varepsilon }(t-\tau )\right] |u-v|_{L^{1}(\Omega )}dt\ge 0,$$

Let $$\varepsilon \rightarrow 0$$. Then

$$|u(x,\tau )-v(x,\tau )|_{L^{1}(\Omega )}\le |u(x,s)-v(x,s)|_{L^{1}(\Omega )}+c\cdot \text {essup}_{\Sigma _{2}\times (0,T)}|f(x,t)-g(x,t)|$$

and the desired result follows by letting $s \rightarrow 0$.

## On the half space

At the last section of the paper, let’s consider Eq. (1) on the half space

43 $$\frac{\partial u}{\partial t} =\Delta A(u)+\text {div}(b(u)),\ (x,t)\in \mathbb {R}^{N}_{+} \times (0,T),$$

with the initial value condition

44 $$u(x,0)=u_0(x), x\in \mathbb {R}^{N}_{+},$$

where $\mathbb {R}^{N}_{+}=\{x=(x_1, x_2, \ldots , x_N): x_N>0\}$ is the half space of $\mathbb {R}^{N}$. The author Zhan (2015b) had shown that when $b_{N}'(0)<0$, we can give Direchlet homogeneous boundary value

45 $$u(x,t)=0,\ (x,t)\in \partial \mathbb {R}^{N}_{+} \times (0,T)=\Sigma \times (0,T).$$

while $b_{N}'(0)\ge 0$, no any boundary value condition is necessary. Now, we give the definitions of the entropy solutions of the Eq. (43), which are the minor versions of the Definition 2.1 in Zhan (2015b).

### **Definition 2**

Let $b_{N}'(0)<0$. A function *u* is said to be the entropy solution of Eq. (43) with the initial value (44) and the boundary value (45), if

1. *u* satisfies
  $$u\in BV(Q_{T})\cap L^{\infty }(Q_{T})\cap L^{1}(Q_{T}),\;\frac{\partial }{\partial x_{i}}\int _{0}^{u}\sqrt{a(s)}ds\in L^{2}(Q_{T}).$$
2. For any $\varphi$, $\varphi \in C_{0}^{2}({Q}_{T})$, $\varphi \ge 0$, for any $k\in \mathbb {R}$, for any small $\eta >0$, *u* satisfies
  46 $$\iint\nolimits _{Q_{T}}\left[ I_{\eta }(u-k)\varphi _{t}-B_{\eta}^{i}(u,k)\varphi _{x_{i}}+A_{\eta }(u,k)\Delta \varphi -S_{\eta}^{\prime }(u-k)\left| \nabla \int_{0}^{u}\sqrt{a(s)}ds\right|^{2}\varphi \right] dxdt \ge 0.$$
3. For any positive constant *R*,
  47 $$\ \ \ \underset{t\rightarrow 0}{\lim }\int _{ B_R(x^R)}\left|u(x,t)-u_{0}(x)\right| dx=0.\;$$
  for any given positive constant *R*, where $x^R=(0,0,\ldots , 0, R)$, $B_R(x^R)=\{x\in \mathbb {R}^{N}_{+}: |x-x^R|<R\}$.
4. The boundary value condition (45) is true in the sense that the traces $\gamma u=\gamma v=0$ on $\partial \mathbb {R}^{N}_{+}$ as usual.

### **Definition 3**

Let $b_{N}'(0)\ge 0$. A function *u* is said to be the entropy solution of Eq. (43) with the initial value (44), if *u* satisfies Definition  2 except the fourth point. In this case, no boundary value condition is required.

Now, we actually are able to prove the existence of the solutions defined as Definition 2–3 in a similar way as Zhan (2015b), we omit the details here. In what follows, we only provide a new and simpler proof of the stability of the solutions.

### **Theorem 2**

*Suppose that* *A*(*s*) *and* $b_{i}(s)$ *are smooth enough. Let* *u*, *v* *be solutions of Eq.* (1) *with the different initial values* $u_{0}(x)$, $v_{0}(x)\in L^{\infty }(\mathbb {R}^{N}_{+})\bigcap L^{1}(\mathbb {R}^{N}_{+})$ *respectively. If* $b_{N}'(0)<0$, *suppose that the traces* $\gamma u=\gamma v=0$ *on* $\Sigma$ *as* (45). *Then*

48 $$\int _{\mathbb {R}^{N}_{+}}\left|u(x,t)-v(x,t)\right| dx\le \int _{\mathbb {R}^{N}_{+}}\left|u_{0}-v_{0}\right| dx.$$

*If* $$b_{N}'(0)\ge 0$$, *then*

49 $$\int _{\mathbb {R}^{N}_{+}}\left|u(x,t)-v(x,t)\right| dx\le \int _{\mathbb {R}^{N}_{+}}\left|u_{0}-v_{0}\right| dx+c\text {esssup}_{\Sigma \times (0,T)}|u(x,t)-v(x,t)|.$$

### *Proof*

By Kruzkov’s bi-variables method, as we have done in section “Kruzkov’s bi-variables method”, we can get

50 $$\iint_{Q_{T}}\left[ \left| u(x,t)-v(x,t)\right| \phi _{t}-\text {sgn} (u-v)(b_{i}(u)-b_{i}(v))\phi _{x_{i}}+ |A(u)-A(v)|\Delta \phi \right] dxdt\ge 0.$$

Now, we can choose $\phi$ in (50) by

$$\phi (x,t)=\omega _{\lambda }(x)\eta (t),$$

where $\eta (t)\in C_{0}^{\infty }(0,T)$, and $\omega _{\lambda }(x)\in C_{0}^{2}(\Omega )$ is defined as follows: for any given small enough $0<\lambda$, $0\le \omega _{\lambda }\le 1$, $\omega |_{\partial \Omega }=0$, and when $x_{N}\ge \lambda$,

$$\omega _{\lambda }(x)=1,$$

when $$0\le x_{N}\le \lambda$$,

$$\omega _{\lambda }(x)=\omega _{\lambda }(x_{N})=1-\frac{(x_{N}-\lambda )^{2}}{\lambda ^{2}}.$$

Clearly,

$$\phi _{x_{i}}= \eta (t)(\omega _{\lambda }(x_{N}))_{x_{i}}\le c|\omega '_{\lambda }(x_{N})|\le \frac{c}{\lambda }. \triangle \phi = \eta (t)\triangle (\omega _{\lambda }(x_{N})) = \eta (t)\nabla (\omega '_{\lambda }(x_{N})\nabla x_{N}) = -\eta (t)\frac{2}{\lambda ^{2}}.$$

Then by (50),

51 $$0 \le \iint_{Q_{T}}\left| u(x,t)-v(x,t)\right| \eta '(t)\omega _{\lambda }(x)dxdt\nonumber -\iint_{Q_{T}}\left[ \text {sgn} (u-v)(b_{N}(u)-b_{N}(v))\eta (t)\frac{\partial \omega _{\lambda }(x)}{\partial x_{N}}+ |A(u)-A(v)|\eta (t)\Delta \omega _{\lambda }(x)\right] dxdt \le \iint_{Q_{T}}\left| u(x,t)-v(x,t)\right| \eta '(t)\omega _{\lambda }(x)dxdt +c\int _{0}^{T}\eta (t)dt\frac{1}{\lambda }\int _{\{x_{N}<\lambda \}} |u-v|dx.$$

Noticing $\lim _{\lambda \rightarrow 0}\omega _{\lambda }=1$, by the definition of the trace of a BV function, let $\lambda \rightarrow 0$ in (51). Then

52 $$\iint_{Q_{T}}\left| u(x,t)-v(x,t)\right| \eta '(t)dxdt +c\text {essup}\left|u-v\right| _{\Sigma \times (0,T)}\ge 0.$$

Let $$0<s<\tau <T$$, and

$$\eta (t)=\int _{\tau -t}^{s-t}\alpha _{\epsilon }(\sigma )d\sigma ,\;\ \epsilon <\min \{\tau , T-s\},$$

as before. Let $\epsilon \rightarrow 0$. Then

$$\int _{\Omega }|u(x,s)-v(x,s)|dx\le \int _{\Omega }|u(x,\tau )-v(x,\tau )|dx+c\text {essup}|\left|u-v\right|_{\Sigma \times (0,T)}.$$

Let $s \rightarrow 0$. If $b_{N}'(0)<0$, suppose that the traces $\gamma u=\gamma v=0$ on $\Sigma$,

53 $$\int _{\mathbb {R}^{N}_{+}}\left|u(x,t)-v(x,t)\right| dx\le \int _{\mathbb {R}^{N}_{+}}\left|u_{0}-v_{0}\right| dx.$$

If $b_{N}'(0)\ge 0$, no any boundary value condition is necessary, then

54 $$\int _{\mathbb {R}^{N}_{+}}\left|u(x,t)-v(x,t)\right| dx\le \int _{\mathbb {R}^{N}_{+}}\left|u_{0}-v_{0}\right| dx+c\text {esssup}_{\Sigma \times (0,T)}|u(x,t)-v(x,t)|.$$

we have the conclusion.

## Conclusion

The paper shows that there is an essential difference of the boundary conditions between the strongly degenerate parabolic equation and the weakly degenerate parabolic equation. Instead of the whole boundary $\partial \Omega$, only a part of $\partial \Omega$ on which we can impose the boundary value if the well-posedness of the solutions to a strongly parabolic equation is considered. In physics, for example, if we consider a special case of Eq. (1), we consider the nonlinear heat conduction equation

$$u_{t}=\text {div}(k(u)\nabla u),$$

if $k(0)=0$, it means there is not heat flux across the boundary. Then the partial boundary $\Sigma _{1}=\emptyset$, so there is no any boundary condition is necessary.
